# Biobased Dyes as Conductive Additives to Reduce the Diameter of Polylactic Acid Fibers during Melt Electrospinning

**DOI:** 10.3390/ma13051055

**Published:** 2020-02-27

**Authors:** Kylie Koenig, Naveen Balakrishnan, Stefan Hermanns, Fabian Langensiepen, Gunnar Seide

**Affiliations:** Aachen-Maastricht Institute for Biobased Materials (AMIBM), Maastricht University, Brightlands Chemelot Campus, Urmonderbaan 22, 6167 RD Geleen, The Netherlands; kylie.konig@maastrichtuniversity.nl (K.K.); naveen.balakrishnan@maastrichtuniversity.nl (N.B.); stefan.hermanns@maastrichtuniversity.nl (S.H.); gunnar.seide@maastrichtuniversity.nl (G.S.)

**Keywords:** fiber spinning, alizarin, quercetin, hematoxylin, crystallinity, nanotechnology

## Abstract

Electrospinning is widely used for the manufacture of fibers in the low-micrometer to nanometer range, allowing the fabrication of flexible materials with a high surface area. A distinction is made between solution and melt electrospinning. The former produces thinner fibers but requires hazardous solvents; whereas the latter is more environmentally sustainable because solvents are not required. However, the viscous melt requires high process temperatures and its low conductivity leads to thicker fibers. Here, we describe the first use of the biobased dyes alizarin; hematoxylin and quercetin as conductive additives to reduce the diameter of polylactic acid (PLA) fibers produced by melt electrospinning; combined with a biobased plasticizer to reduce the melt viscosity. The formation of a Taylor cone followed by continuous fiber deposition was observed for all PLA compounds; reducing the fiber diameter by up to 77% compared to pure PLA. The smallest average fiber diameter of 16.04 µm was achieved by adding 2% (*w*/*w*) hematoxylin. Comparative analysis revealed that the melt-electrospun fibers had a low degree of crystallinity compared to drawn filament controls—resembling partially oriented filaments. Our results form the basis of an economical and environmentally friendly process that could ultimately, provide an alternative to industrial solution electrospinning

## 1. Introduction 

Electrospinning is a simple, versatile and cost-effective method to produce fibers in the low-micrometer to nanometer range, thus making a significant contribution to the booming nanotechnology industry [1,2,3]. The beneficial properties of such fibers include their flexibility and enormous surface area, leading to applications in medicine [4,5,6,7,8], filtration and separation [9,10], electronics and energy [11,12], and textile manufacturing [13,14,15]. 

Electrospinning involves the exposure of liquids to strong electric fields. When a large potential difference (tens of kilovolts) is applied to a liquid flowing through a capillary, the liquid forms a jet that may undergo whip-like movements, stretching the fluid and yielding microscale or nanoscale fibers that are deposited on a collector [15]. The two principal types of electrospinning are solution electrospinning, where the polymer is dissolved in a solvent that evaporates to produce the fibers, and melt electrospinning, where a molten polymer is cooled to produce the fibers [1]. Solution electrospinning is easier to implement, has been studied more widely, and is favored by industry, despite the environmental hazards posed when toxic solvents are required. In contrast, research and technology uptake in the field of melt electrospinning has been held back by complex equipment requirements [16], the problem of electric discharge [17], and the high-temperature, high-viscosity and low-conductivity of the polymer melt [18]. Accordingly, parameters that affect the viscosity, conductivity, thermal and structural properties of solution-electrospun fibers are well understood, allowing the production of finer fibers, whereas equivalent studies focusing on melt electrospinning are still at an early stage [1,19]. 

The wider adoption of melt electrospinning could help to reduce the environmental footprint of current industrial electrospinning processes, which require an expensive solvent recovery process and present a high risk of toxic solvent carryover into the final product. For example, one of the commonly used materials in the industrial electrospinning process is polylactic acid (PLA) because it is a sustainable polymer made from renewable agricultural resources and is reported to be industrially compostable. Commercial low-molecular-weight PLA was reported to have a biodegradation degree of 72% after 110 days under aerobic conditions [20]. However, PLA sub-microfibers are usually prepared using the toxic solvents dichloromethane, chloroform or N,N-dimethylformamide [21]. It is, therefore, desirable to improve melt-electrospinning technology, aiming to reduce the fiber diameter by overcoming the limitations described above. One promising approach is the use of additives to increase the conductivity of polymer melts, and plasticizers to reduce their viscosity, in order to produce thinner fibers [22,23,24]. PLA is the most commercially available biobased and biodegradable thermoplastic polymer with increasing use in the textile sector to replace petroleum-based polymers [25]. It is, therefore, an important substrate for melt-electrospinning technology. PLA fibers produced by melt electrospinning have been modified by adding a plasticizer [21,26], and by adjusting the device during fabrication to facilitate airflow [27] or incorporate laser heating [17]. These approaches led to the production of fibers with diameters in the range 0.2–50 µm [17,18,21,27,28,29,30,31,32,33]. However, most of the additives used thus far are unsustainable chemicals that offset the environmental advantages of biobased polymers. They are difficult to disperse in the polymer melt, or they adsorb water and, therefore, interfere with high-temperature melt-electrospinning processes [21,26,34]. 

**Figure 1:**
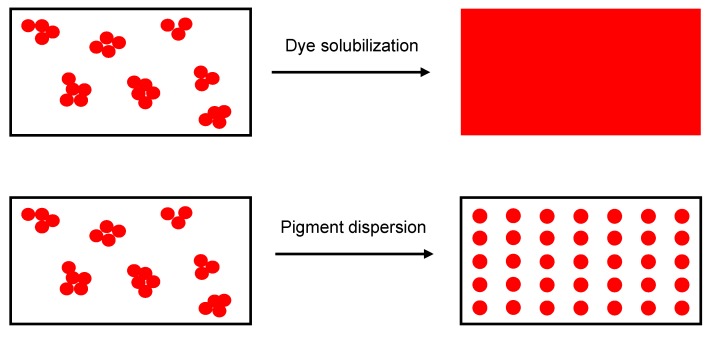
Schematic representation of dye solubilization and pigment dispersion.

Sustainable colorants are used extensively in the textile industry, offering a promising alternative to conventional additives [35]. Colorants can be classified as dyes or pigments. Dyes are molecules that can be solubilized in a polymer substrate, they have good chemical affinity for the polymer, and therefore, retain transparency; in contrast, pigments are insoluble in the polymer substrate and are dispersed as very fine particles (Figure 1). Common organic textile dyes include alizarin and curcuma, whereas common pigments include copper phthalocyanine and carbon black [36]. Colorants such as alizarin, purpurin and phthalocyanine have already been used to manufacture electronics such as field effect transistors and dye-sensitized solar cells [37] because the presence of functional groups and/or π-conjugation improves conductivity, and charge carrier mobility may exceed 1 cm^2^/Vs [38].

The aim of this study was to investigate the ability of three biobased dyes to increase the conductivity of molten PLA during melt electrospinning, combined with a biobased plasticizer to reduce melt viscosity for an overall reduction of the obtained fiber diameters. We selected the biobased dyes alizarin, quercetin and hematoxylin, which have not previously been used in a melt-electrospinning process, and determined their effect on the diameter of PLA fibers manufactured using a single-nozzle melt-electrospinning device. We compared fibers incorporating different weight percentages of dye with or without the biobased plasticizer. We determined the influence of the additives on viscosity, conductivity, degradation and thermal behavior. We also compared the morphology and crystallization behavior of melt-electrospun fibers to melt-spun fibers with different draw ratios. Our results can be used to develop an environmentally beneficial melt-electrospinning process for the manufacture of microscale and nanoscale fibers.

## 2. Materials and Methods

### 2.1. Materials

PLA grade L130 (Total|Corbion, Gorinchem, The Netherlands) was used as the base polymer for all experiments. The following specifications were reported by the manufacturer: L-content ≥ 99%, glass transition temperature (T_g_) ~60 °C, and melt flow index = 24 g/10 min at 210 °C/2.16 kg. The chemical structures and melting points of the biobased dyes alizarin, quercetin, and hematoxylin (Sigma-Aldrich, Zwijndrecht, The Netherlands) are presented in Table 1. Liquid dyes (Rowasol, Pinneberg, Germany) were prepared by stirring 25% (*w*/*w*) of the biobased dyes with a plasticizer based on vegetable oil. The dyes were used without plasticizer to test their effect on melt conductivity and with plasticizer to determine the combined effect of increasing the conductivity and reducing the viscosity of the melt.

### 2.2. Micro-Compounder

PLA was vacuum-dried at 80 °C overnight before compounding. PLA compounds were made by mixing with 1% or 2% (*w*/*w*) of each additive in a micro-compounder (Xplore, Sittard, The Netherlands) at 200 °C with a screw speed of 100 rpm for 2 min. The list of compounds and their abbreviations are presented in Table 2.

### 2.3. Melt-Spinning Equipment

Partially oriented filaments (PFs) were prepared using a KETSE 20/40 twin-screw extruder (Brabender, Duisburg, Germany) with a spinning head. Melt spinning was performed at 200 °C using a spinneret with 24 holes, each 0.4 mm in diameter, and a length to diameter ratio of two. A constant throughput was maintained and the multi-filaments were wound at 150 m/min. The PFs were post-drawn at 100 °C with a draw ratio of 5 to obtain drawn filaments (DFs). The crystallinity of these filaments was compared to the melt-electrospun fibers.

### 2.4. Melt-Electrospinning Equipment

**Figure 2:**
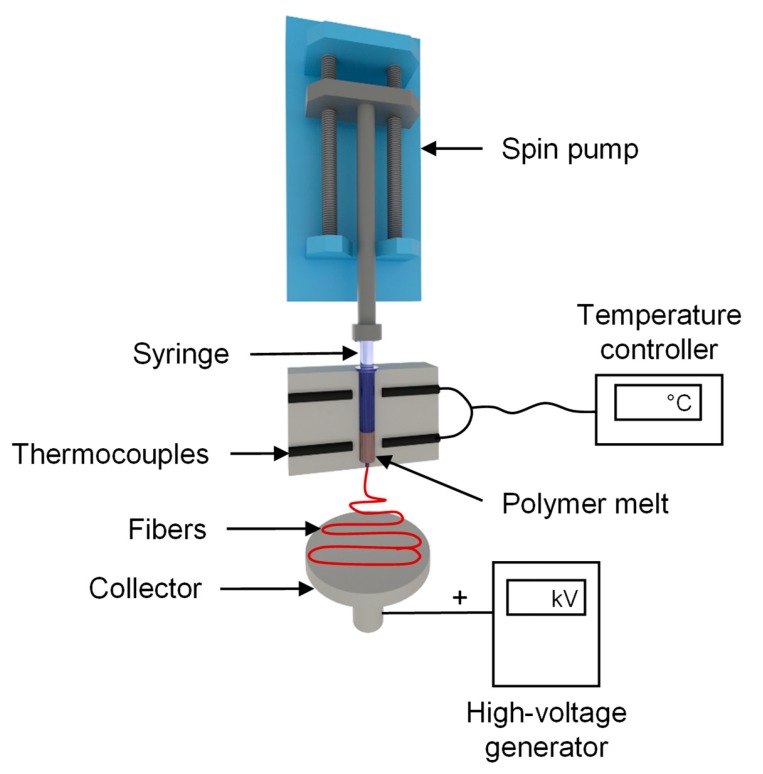
Laboratory single-fiber melt-electrospinning setup.

We used a self-configured laboratory-scale single-fiber melt-electrospinning device consisting of five major components: temperature controller, high-voltage power supply, heating elements, syringe pump, and collector (Figure 2). The device was equipped with JCS-33A temperature process controllers (Shinko Technos, Osaka, Japan) and PT 100 platinum thermocouples (Omega Engineering, Deckenpfron, Germany) to control the melting temperature. The temperature was set to 275 °C for the pure polymer and polymer with additives. A KNH65 high-voltage generator (Eltex-Elektrostatik, Weil am Rhein, Germany) with a voltage range of 6–60 kV was used. During the melt-electrospinning experiments, the voltage was kept constant at 50 kV. A positive voltage was applied to the collector while grounding the spinneret. A flat aluminum plate (6 cm) overlaid with a thin paperboard was used as a collector. The distance between the spinneret and collector was set at 10 cm for all trials. An 11 Plus spin pump (Harvard Apparatus, Cambridge, MA, USA) was used with a constant delivery rate of 4 mL/h. A 2-mL glass syringe (Poulten & Graf, Wertheim, Germany) with a nozzle orifice of 1 mm served as the spinneret. 

### 2.5. Characterization of Compounds

Differential scanning calorimetry (DSC) was performed using a Q2000 device (TA Instruments, New Castle, DE, USA) to determine the influence of the additive on the different thermal transition temperatures of PLA. The tests were carried out at a heating rate of 10 °C/min between 25 and 200 °C with a sample size of ~5 mg. The parameters were kept constant for all samples to ensure comparability. TA universal analysis software was used to visualize and compare the data. We compared the T_g_, cold crystallization temperature (T_cc_), melting point (T_m_), and percentage crystallinity (X_c_). The melt enthalpy of 100% crystalline PLA was considered to be 93.7 J/g [39]. All compounds were prepared using the same protocol, so any differences in material properties should primarily reflect the nature and quantity of additives.

A Q5000 device (TA instruments) was used to carry out thermogravimetric analysis (TGA) at a heating rate of 10 °C/min under nitrogen flow up to 500 °C. The temperatures at 5% and 50% weight loss were determined using TA universal analysis software and the values were compared to determine the influence of additives on the thermal stability of PLA.

Rheological characterization was carried out using a Discovery HR1 hybrid rheometer (TA Instruments). We performed one flow sweep using a 25 mm plate with an increasing shear rate (0.01–500 rad/s). The gap between the plates was maintained at 1000 µm, and the strain amplitude and environment temperature were maintained at 0.5% and 200 °C, respectively. For better comparability, the viscosity of the pure PLA and all the compounds are presented at a shear rate of 5 rad/s. 

The TGA experiment was not isothermal and could only determine the weight loss, so we also measured actual degradation in terms of molecular weight after compounding and melt electrospinning by gel permeation chromatography (GPC) using a 1260 Infinity System (Agilent Technologies, Santa Clara, CA, USA). We used hexafluor-2-isopropanol (HFIP) containing 0.19% sodium trifluoroacetate as the mobile phase at a flow rate of 0.33 mL/min. Solutions were prepared by dissolving 5 mg of pure PLA and the various compounds in HFIP for ~2 h, passing the solutions through a 0.2 µm polyetrafluoroethylene filter, and injecting them into a modified silica column filled with 7 µm particles (Polymer Standards Service, Mainz, Germany). The experiment was calibrated against a standard polymethyl methacrylate polymer (1.0 × 10^5^ g/mol) and the relative molecular weight (M_w_), number average molar mass (M_n_), and polydispersity index (PDI) of each polymer were recorded and compared.

**Figure 3:**
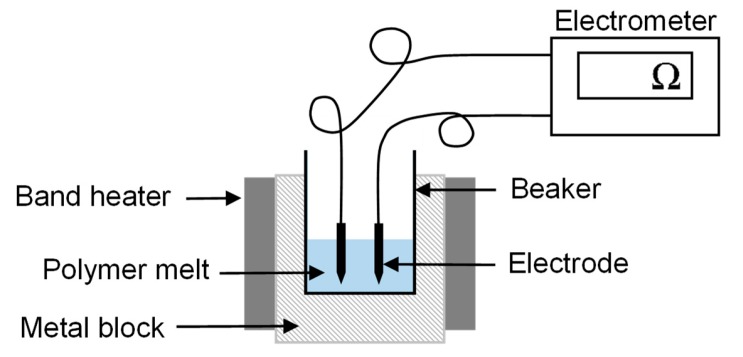
Configuration used for the measurement of electrical resistance.

The electrical resistance of the pure polymer and compounds was measured at an elevated temperature of 325 °C using a Keithley 617 electrometer (Tektronix Inc., Beaverton, OR, USA) as shown in Figure 3. The experimental setup has been previously used for conductivity measurements of polymer melts [19]. The polymer granulate was melted using band heaters, and two electrodes, 6 mm apart, were dipped in the melt and connected to the electrometer. The electrical current flowing between the electrodes was measured by applying a constant 10 V. 

### 2.6. Characterization of the Fibers

Fiber diameters were determined by reflected light microscopy using a DM4000 M instrument (Leica Microsystems, Wetzlar, Germany) at 100–200× magnification, and images were captured using Leica Application Suite software. Ten images representing different areas of each non-woven fiber were used to determine the average fiber diameter. DSC was performed on all melt-electrospun fibers to determine the effect of additives and the electrospinning process. DSC was also carried out on the PFs and DFs, as well as the melt-electrospun PLA fibers under the same testing conditions. The thermal transition temperatures and X_c_ values were compared. Polarized optical microscopy (POM) was used to investigate the crystallinity of the melt-electrospun filaments, PFs and DFs. An Olympus BX53 microscope and DP26 camera (Olympus BV, Leiderdorp, The Netherlands) were used to capture the images at 50× magnification. The images were screened for birefringence. The relationship between the electrical resistance, melt viscosity, and average fiber diameter was visualized in surface plots using Minitab19 analysis software.

### 2.7. Cost Analysis

Only 2%–10% of the liquid processed during solution electrospinning is the polymer (the rest is solvent that evaporates), whereas 100% of the processed liquid solidifies into fibers during melt electrospinning [1]. The cost of PLA is 2–4 €/kg depending on the grade, and we used a nominal value of 2.1 €/kg in our cost model. In solution electrospinning, PLA is prepared as a 10% solution in chloroform, which costs ~100 €/liter. Accordingly, 10 L of chloroform is required to make 1 kg of PLA fiber. The total material cost of 1 kg of PLA fiber is, therefore, ~1000 € for the first production cycle without recovery of the solvent and solvent disposal according to standards. For an off-site solvent recovery, the cost is estimated to be ~100 € per 200 L of solvent [40]. Considering the case of production of 1 kg of PLA fiber, when 90% of the solvent used can be recovered, 9 L of the solvent can be reused and it would cost ~4.5 €. For the remaining 10%, the cost of purchasing new solvent would still be ~100 € and the overall solvent cost would be more than 100 €. In contrast, organic dyes as conductive additives are much less expensive. For example, alizarin costs ~900 €/kg and 1 kg of PLA fiber containing 2% (*w*/*w*) of this dye would cost 3 €. Therefore, using organic dyes not only makes the process more sustainable, it is also economical.

### 2.8. Methodology

**Figure 4:**
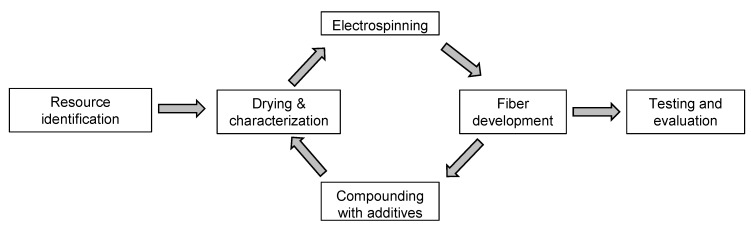
Overview of the experimental workflow.

Our overall workflow is summarized in Figure 4. The PLA was characterized and dried before melt electrospinning, and the diameter of the melt-electrospun pure PLA fiber was measured. We then tested various combinations of additives (dyes with or without plasticizer) to reduce the viscosity of the melt and increase its conductivity, in order to produce thinner fibers. We prepared 12 compounds in total (Table 2) representing each of the three dyes at concentrations of 1% *w*/*w* (A1, H1 and Q1) and 2% *w*/*w* (A2, H2 and Q2), as well as the liquid dyes in plasticizer also at additive concentrations of 1% *w*/*w* (LA1, LH1 and LQ1) and 2% *w*/*w* (LA2, LH2 and LQ2). The compound names were based on the initial letter of each dye: A = alizarin, H = hematoxylin and Q = quercetin, with L referring to the liquid form. Melt electrospinning was carried out with the compounds and the diameter of the resulting fibers was measured. All other process parameters (e.g., temperature, throughput, electric field strength) were kept constant. 

## 3. Results and Discussion

### 3.1. Thermal Properties of the PLA Compounds

**Figure 5:**
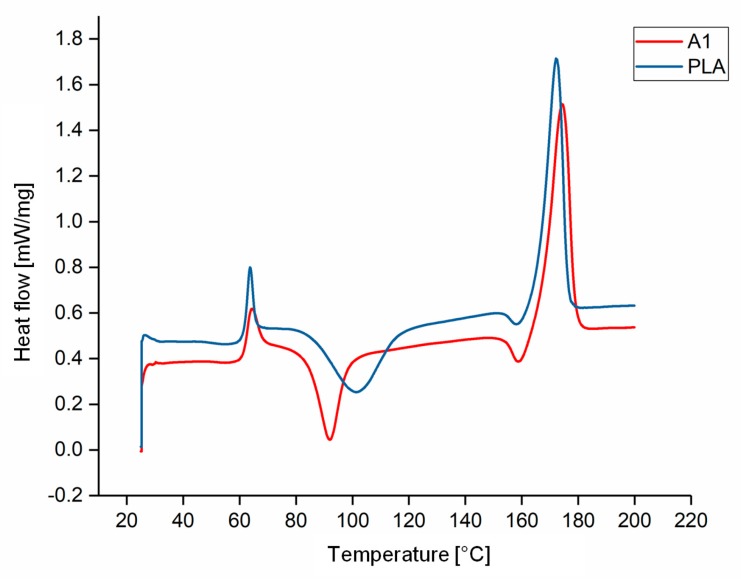
DSC thermogram of PLA and compound A1.

**Figure 6:**
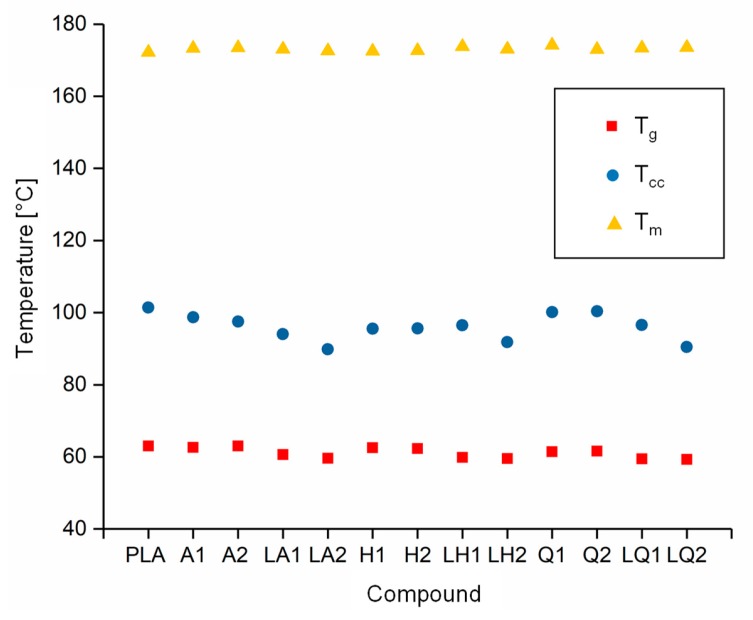
Thermal transition temperature of PLA and the PLA/dye compounds.

**Figure 7:**
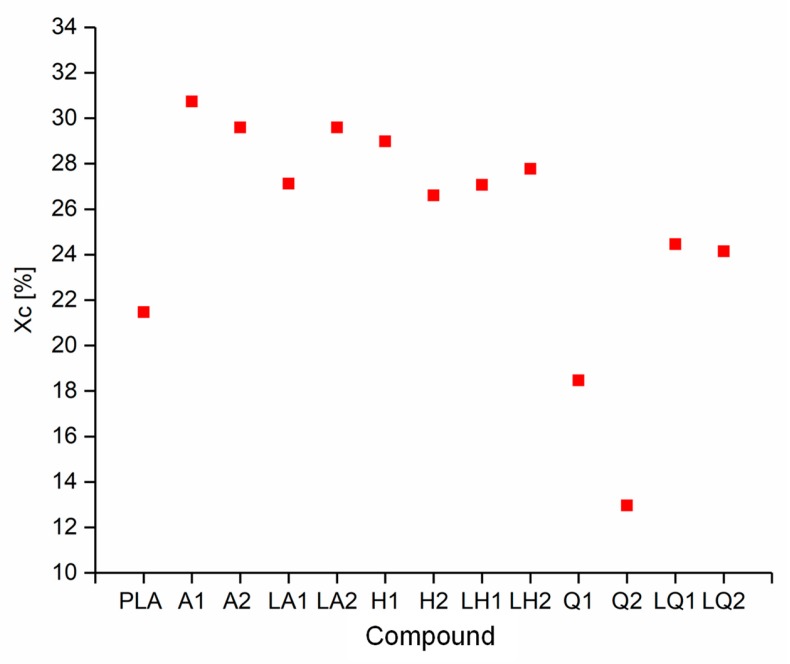
Crystallinity (X_c_) of PLA and the PLA/dye compounds.

The DSC thermograms of PLA and A1 are compared in Figure 5 as a representative example of the experiments because all the compounds behaved in a similar manner. The T_g_, T_cc_, T_m_ and X_c_ values of each compound are compared visually in Figure 6 and Figure 7. The T_g_ and T_m_ did not change significantly and remained at ~60 °C and ~173 °C, respectively, regardless of the additive and weight percent. The temperature values for PLA are consistent with those previously reported in the literature [41,42]. In contrast, the additives had a significant effect on T_cc_. The T_cc_ of PLA was ~101 °C but this declined to 91.30 °C for A1 and 87.20 °C for A2. Furthermore, the X_c_ of PLA was 21.47%, but this increased to 30.72% for A1 and 29.60% for A2. The X_c_ of the liquid alizarin compounds also increased compared to pure PLA. Although the T_cc_ of the hematoxylin and liquid hematoxylin compounds was not much lower than the value for pure PLA, the X_c_ of these compounds increased compared to pure PLA. There are two possible explanations for this behavior observed in the cases of these compounds. First, the dye may induce nucleation, as previously reported for polypropylene samples containing colorants [43,44]. Second, the dye may trigger the degradation of PLA, leading to shorter and more mobile polymer chains that are more likely to undergo crystallization. This was explored by GPC analysis (Section 3.3). The T_cc_ of compounds Q1 and Q2 was similar to that of PLA, but the X_c_ decreased from 21.47% to 18.47% and 12.97%, respectively. Furthermore, in the case of quercetin, two possible explanations should be considered. The first is that the added quercetin affects the chain mobility and thus disturbs the crystallization process. Similar observations were reported when adding nigrosine dye to polyamide 66 [45]. In case of LQ, since the overall content of quercetin is lower, the hindrance to crystallization is also lower. The second possible explanation is the degradation theory as explained earlier in the case of hematoxylin. This was also explored by GPC analysis (Section 3.3).

**Figure 8:**
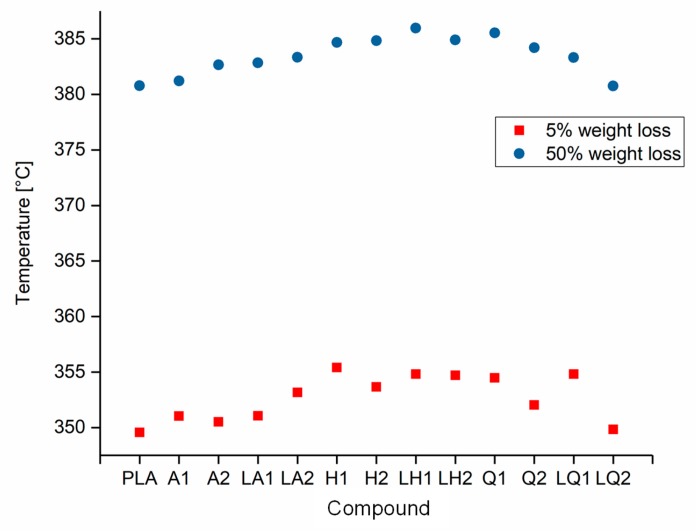
Temperature at 5% and 50% weight loss of PLA and the PLA/dye compounds.

The decomposition temperatures of pure PLA and its compounds were determined by TGA. The temperatures at which 5% and 50% weight loss occurred are compared in Figure 8. The maximum 5% weight loss temperature was observed for compound LH1 at 355 °C and the minimum was observed for pure PLA at 350 °C. Similarly, the maximum 50% weight loss temperature was observed for compound LH1 at 386 °C and the minimum was observed for pure PLA at 381 °C. There was no significant difference in the temperature range over which these weight losses occurred. Since the TGA could only measure the weight loss in the form of the released volatile gases, we characterized the degradation leading to molecular weight reduction and oligomer formation by GPC (Section 3.3) and rheological analysis (Section 3.2).

### 3.2. Effects of Additives on Melt Viscosity

**Figure 9:**
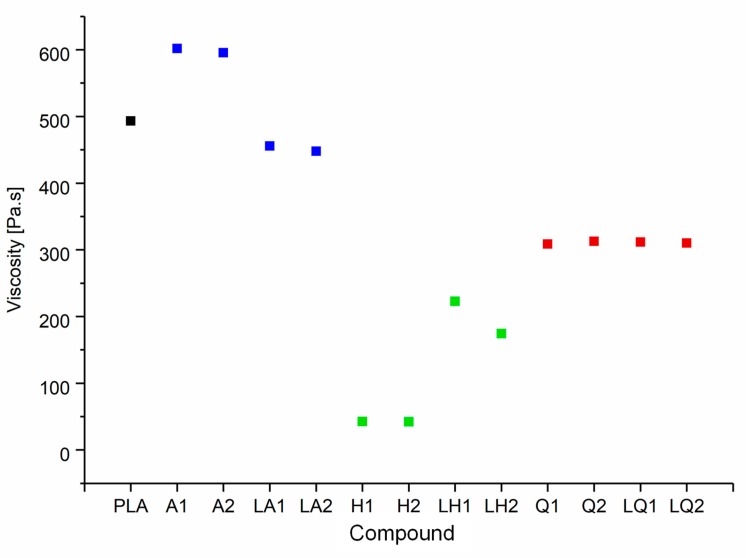
Shear viscosity of pure PLA and the PLA/dye compounds, corresponding to a set temperature of 200 °C and a shear rate of 5 rad/s.

The shear viscosities of pure PLA and its compounds at a set temperature of 200 °C and a shear rate of 5 rad/s are summarized in Figure 9. The viscosity of the polymer melt increased by ~22% following the addition of alizarin, from 493 Pa·s (PLA) to 601 Pa·s (A1) and 595 Pa·s (A2). Higher melt viscosity tends to increase fiber diameters during melt electrospinning, but narrower fibers can still be achieved if the additives increase the electrical conductivity of the melt [19]. The addition of quercetin had a plasticizing effect on the PLA and reduced the melt viscosity by ~37%, from 493 Pa·s (PLA) to 308 Pa·s (Q1) and 312 Pa·s (Q2). However, the addition of hematoxylin achieved the most dramatic effect, reducing the melt viscosity by ~91% at both concentrations, to 42 Pa·s. The melting point of hematoxylin (200 °C) is much lower than that of the other dyes, so the low viscosity of the compounds containing hematoxylin may reflect the melting of the dye along with the polymer. The other possible hypothesis for reducing the viscosity of both hematoxylin and quercetin compounds is polymer degradation. The degradation hypothesis was addressed by GPC analysis (Section 3.3). 

Overall, there was little difference in viscosity between compounds containing 1% or 2% (*w*/*w*) of a given dye. This was not the case for the liquid dyes, where the plasticizer showed a significant concentration-dependent effect. In the case of alizarin, the dye itself increased the melt viscosity, whereas the plasticizer has the opposite effect; therefore, the combination showed only a slight reduction in viscosity compared to pure PLA (7% for LA1 and 9% for LA2). In contrast, because hematoxylin and quercetin reduced the melt viscosity, the addition of plasticizer was expected to enhance this effect. Surprisingly, this was not the case—the viscosity of the liquid hematoxylin and quercetin compounds was significantly higher than the compounds prepared with pure dyes. For example, the viscosity of Q1 was 30% lower than LQ1, and the viscosity of H1 was 81% lower than LH1. Normally the polar group of the plasticizer interacts with the polar group of the polymer, swelling the polymer chains and increasing the free volume. Such interactions would reduce intermolecular cohesion and increase polymer chain mobility, thus reducing the viscosity of the melt [46]. The unexpected behavior of the quercetin and hematoxylin compounds may reflect a stronger interaction between these dyes and the plasticizer compared to the interaction between the polymer and plasticizer. The interaction between dye and plasticizer molecules generates bulky particles that could hinder the motion of the polymer chains and ultimately, increase the viscosity. Since the three dyes used are chemically different, they interact differently with the plasticizer.

### 3.3. Effect of Additives on Polymer Degradation

**Figure 10:**
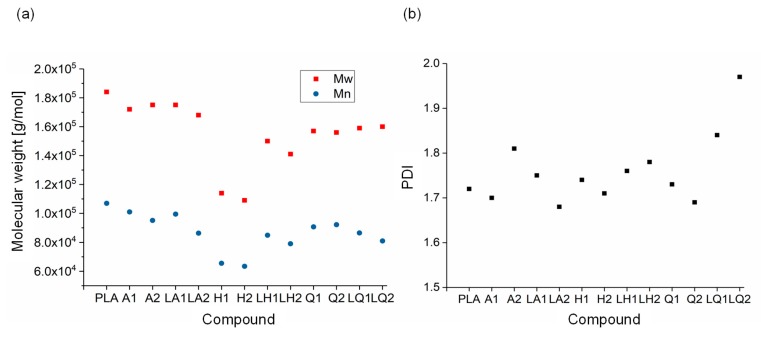
Molecular weight (**a**) and PDI (**b**) of PLA and the PLA/dye compounds.

GPC analysis of PLA and its compounds (Figure 10) revealed M_w_, M_n_ and PDI values of 184,000, 107,000 and 1.72, respectively, for pure PLA. There was no significant change from these values in the alizarin and liquid alizarin compounds. This confirms that the increase in Xc observed for the alizarin and liquid alizarin compounds is a result of the nucleating effect of alizarin. In the quercetin and liquid quercetin compounds, there was a slight reduction in M_w_ and M_n_. The greatest change was observed in compound Q2, where the M_w_ and M_n_ values were 15.12% and 14.01% lower, respectively, compared to those of pure PLA. The PDI increased for both LQ1 and LQ2, and the highest PDI was observed for LQ2. However, since the change in molecular weight is not as drastic as for the hematoxylin compounds, the viscosity of both quercetin and liquid quercetin compounds was observed to be similar. Hence, in this case, the increase in crystallinity of LQ1 and LQ2 can be attributed to the fact that a lower quantity of quercetin was present compared to Q1 and Q2. In the hematoxylin compounds, the changes were more significant. For example, the M_w_ and M_n_ of compound H2 decreased by 40.71% and 40.74%, respectively, compared to pure PLA. As the percentage losses in M_w_ and M_n_ were the same, their PDI ratio remained unaffected. The GPC measurements confirmed that the degradation of compounds containing hematoxylin led to the observed reduction in viscosity and increase in X_c_ (Section 3.2). Although the unexpected increase in viscosity observed following the addition of liquid hematoxylin could be attributed to interactions between the dye and plasticizer, our GPC results provide evidence that the increase in viscosity could also reflect the lower degree of degradation in these compounds.

GPC analysis was also carried out on the melt-electrospun fibers, but no significant reduction of the molecular weight could be detected due the short dwell time of the polymer melt in the syringe of less than less than thirty seconds.

### 3.4. Effect of Additives on Melt Conductivity

**Figure 11:**
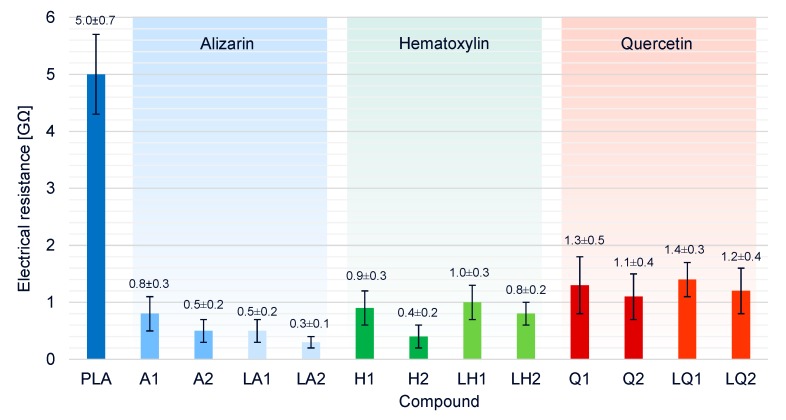
Electrical resistances of pure PLA and PLA/dye compounds.

The electrical resistance of pure PLA its compounds was measured at a set temperature of 325 °C (Figure 11). The higher temperature compared to the spinning process was chosen because more heat energy is lost over the larger surface of the beaker and thus, more energy must be supplied to achieve the same melting conditions. Electrical conductivity requires freely movable charge carriers, so the electrical resistance of pure PLA (5.0 GΩ) decreased by a factor of five in the presence of any of the additives. In the compounds containing dyes but no plasticizer, the electrical resistance was also inversely related to the dye concentration. Because alizarin increased the viscosity of the melt, the higher conductivity is likely to favor the melt-electrospinning process and reduce the fiber diameter. Furthermore, electrical resistance in the liquid alizarin compounds was lower than in compounds containing alizarin but no plasticizer, indicating that the plasticizer also contributes to the higher conductivity, as reported in earlier studies [47,48]. We observed similar behavior in the liquid hematoxylin and liquid quercetin compounds, although the overall effect of quercetin on electrical conductivity was weakest. The synergetic effects of viscosity and conductivity on fiber diameter are described in more detail in Section 3.6. 

### 3.5. Physical Properties of PLA Fibers under Different Processing Conditions

**Figure 12:**
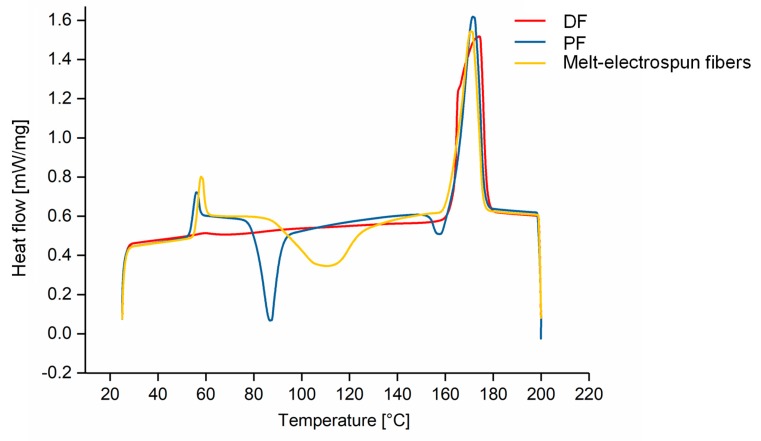
DSC thermograms of partially orientated filaments, drawn filaments and melt-electrospun PLA fibers.

The physical properties of the melt-spun PFs and DFs were compared to the melt-electrospun PLA fibers by DSC (Table 3) and the corresponding thermograms are presented in Figure 12. The T_g_, T_m_ and T_cc_ values were similar for the PFs and melt-electrospun fibers; however, we were unable to determine T_g_ or T_cc_ values after fiber drawing. Furthermore, the X_c_ of the PFs and melt-electrospun fibers were very low (17.93% and 8.72%, respectively), whereas the X_c_ after fiber drawing was 58.83%. The absence of a glass transition in DSC thermograms often occurs when the crystalline fraction is more abundant than the amorphous fraction, and the T_cc_ value is absent because the DF is fully drawn, in agreement with previous studies [49]. The melt-electrospun fibers are, therefore, more similar to a PF than a DF.

**Figure 13:**
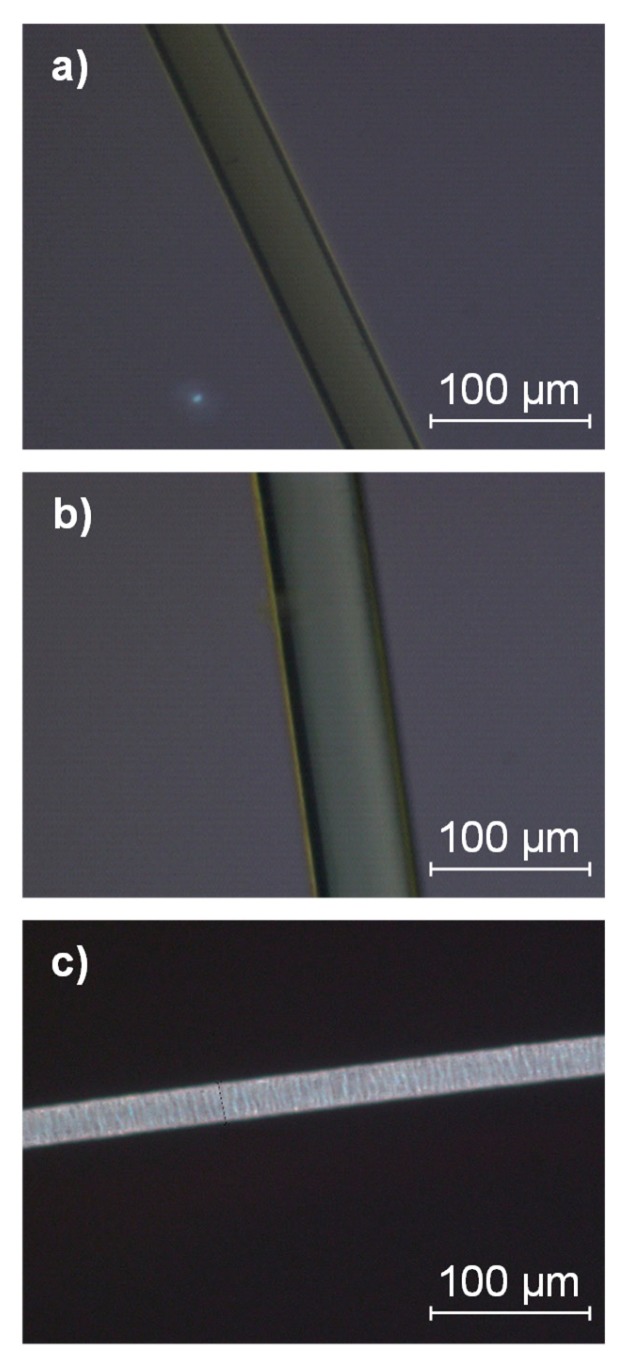
POM images. (**a**) Melt-electrospun PLA fiber. (**b**) Partially oriented PLA filament. (**c**) Drawn PLA filament.

POM analysis of the melt-electrospun fibers, PFs and DFs revealed the extent of crystallinity compared to pure PLA fibers produced under different processing conditions (Figure 13). Optically anisotropic materials such as crystalline materials give rise to birefringence due to the difference in their axis length [50]. Although individual crystals could not be observed by POM, very little to no birefringence was detected in the melt-electrospun fibers and PFs, whereas birefringence was observed in the DFs because the degree of crystallinity was increased by drawing. The same technique was previously used to show that the degree of crystallinity in PA66 tensile bars was dependent on the mold temperature [51]. Our combined DSC and POM data, therefore, indicate that the PLA fibers produced by melt electrospinning are similar to PFs produced by melt spinning, which have a much lower T_cc_ value than DFs.

### 3.6. Fiber Diameters Achieved Using Different PLA Compounds

The processability of the compounds and the influence of additives on the fiber diameter were investigated by producing fibers using a single-fiber melt-electrospinning device. For pure PLA, the formation of a Taylor cone followed by typical fiber deposition was observed at a temperature of 275 °C. Fiber formation was possible with all compounds at this temperature and the corresponding fiber diameters could, therefore, be determined under the same conditions.

**Figure 14:**
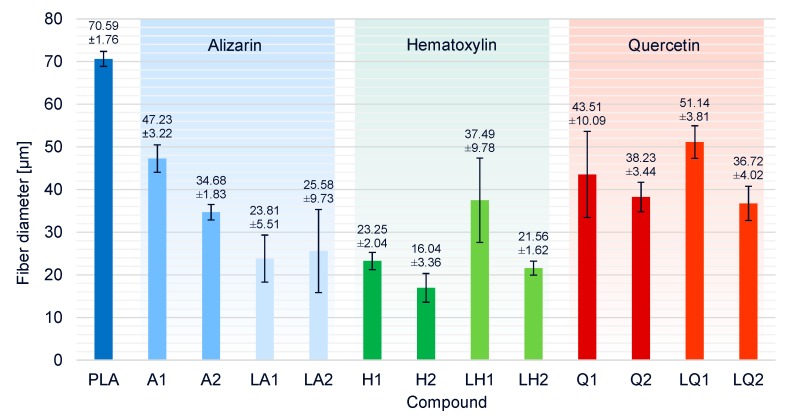
Fiber diameters and standard deviations of PLA and the PLA/dye compounds produced by melt-electrospinning at 275 °C using a single-nozzle laboratory device.

The average fiber diameter for pure PLA was 70.6 µm (Figure 14). All compounds produced thinner fibers, indicating that all the additives affected viscosity and/or conductivity in a beneficial manner. Despite the increase in viscosity caused by the addition of alizarin, the fiber diameters of compounds A1 and A2 were reduced by 33% and 50%, respectively, compared to pure PLA. The increase in electrical conductivity conferred by alizarin, therefore, compensated for the increase in viscosity and the influence of conductivity was dominant, particularly at higher alizarin concentrations. The presence of plasticizer in addition to alizarin reduced the viscosity of the melt, and the smallest fiber diameter of all alizarin compounds was, therefore, achieved by LA1 (23.8 µm, 63% narrower than pure PLA). There was no significant difference between the fiber diameters of LA1 and LA2.

The addition of hematoxylin led to a significant reduction in viscosity, and the degradation of the polymer (and thus a reduction in M_w_) was detected by GPC. As expected, this resulted in the most profound reduction in fiber diameter among all compounds. The finest fibers (16.04 µm, 77% narrower than pure PLA) were achieved for compound H2. The increase in fiber diameter in the presence of the plasticizer matched the unexpected increase in viscosity of liquid hematoxylin compared to hematoxylin compounds without a plasticizer, and the fall in electrical conductivity due to the overall lower concentration of hematoxylin when the plasticizer was present.

Finally, the effect of quercetin on fiber diameter was similar to that of alizarin when each dye was presented in the absence of plasticizer. Interestingly, the dyes had opposite effects in the presence of plasticizer, with the liquid alizarin compounds LA1 and LA2 reducing the fiber diameter further than compounds A1 and A2, but the liquid quercetin compounds LQ1 and LQ2 producing fibers that were similar in diameter or thicker than those based on compounds Q1 and Q2. As discussed above, this mirrors the opposing effects on viscosity: the plasticizer reduced the viscosity of compounds containing alizarin but increased the viscosity of those containing quercetin. These data also suggest that quercetin has a less significant effect on conductivity than alizarin. 

**Figure 15:**
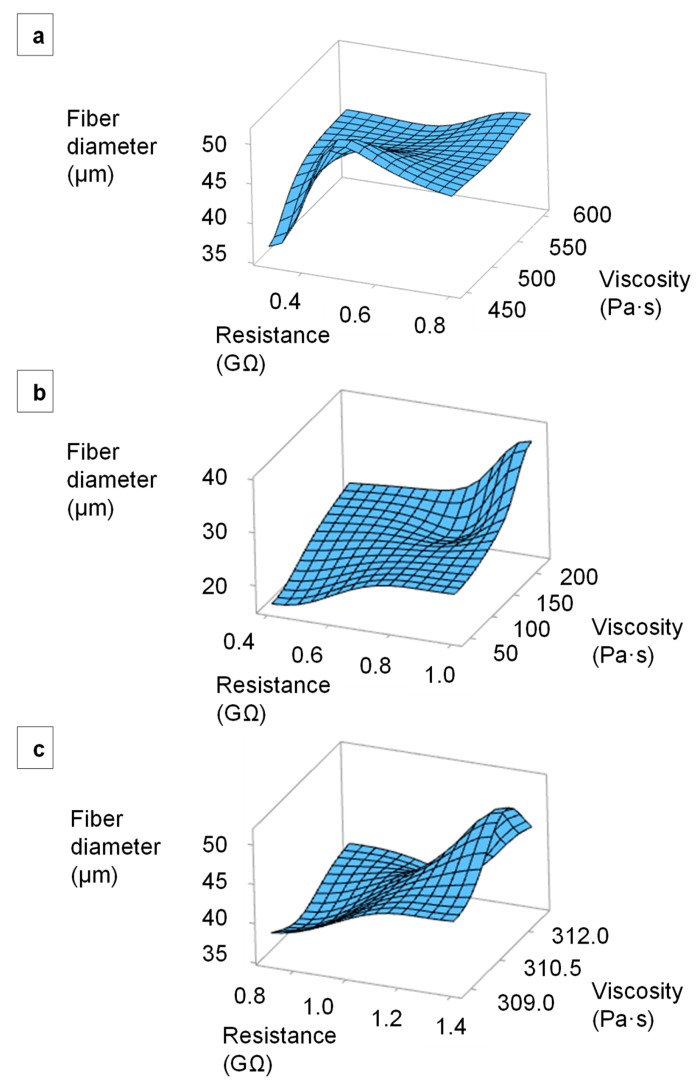
Surface plots of fiber diameters in relation to the electrical resistance and viscosity of the melt. (**a**) PLA/alizarin compounds. (**b**) PLA/hematoxylin compounds. (**c**) PLA/quercetin compounds.

We plotted the relationship between the electrical resistance, melt viscosity and average fiber diameter using Minitab 19 analysis software. Figure 15 presents surface plots of fiber diameters in relation to the electrical resistance and viscosity of the alizarin, hematoxylin and quercetin compounds. It has to be considered that the set temperature of the viscosity and resistance measurement deviates from the set temperature of the spinning process, as explained in 2.5, so that only a trend can be described. As the temperature increases, the viscosity decreases, as well as the resistance [34], so that a further reduction of the fiber diameters is to be expected. The fibers prepared from all three compounds became finer with decreasing melt viscosity and electrical resistance. For the alizarin compounds, the increasing conductivity was the decisive factor controlling fiber diameter because there was little variation in viscosity. The minimum fiber diameter was always achieved using compounds with the lowest viscosity and the lowest electrical resistance.

The fiber diameters we have achieved are still in the micrometer range, like the majority of fiber diameters previously reported in the literature. However, it should be stated that we have not made any device-specific modifications, such as the integration of an accelerated airflow or a heating system as it is already used for other melt-electrospinning systems [27]. Furthermore, the fiber diameter is strongly influenced by the flow rate of the polymer [1], so that the use of a nozzle with a smaller orifice can significantly reduce the fiber diameter in future attempts.

## 4. Conclusions and Further Perspectives

We have successfully tested the biobased dyes alizarin, hematoxylin and quercetin as conductive additives in the melt-electrospinning process, and have produced fibers in the micrometer range. All dyes and dye/plasticizer combinations contributed to the desirable reduction of fiber diameter compared to pure melt-electrospun PLA fibers, which will facilitate the development of an economical and environmentally friendly process for the production of microfibers and nanofibers that could ultimately replace solution electrospinning. The formation of a Taylor cone followed by continuous fiber deposition was observed for all dyes and dye/plasticizer combinations. The finest fibers (16.04 µm in diameter) were produced by adding 2% (*w*/*w*) hematoxylin, reducing the average fiber diameter by 77% compared to pure PLA. However, hematoxylin induced polymer degradation at a spinning temperature of 275 °C, which reduces the M_w_ and, therefore, favors the production of finer fibers. In future experiments, the process temperature should be lowered when using hematoxylin to prevent degradation. The addition of alizarin produced finer fibers than pure PLA despite the increase in melt viscosity, indicating that alizarin has a profound effect on the electrical conductivity of the melt. A combination of alizarin (to increase conductivity) and a plasticizer (to reduce viscosity) reduced the fiber diameter to 23.8 µm, which is 63% narrower than the pure PLA fibers. The addition of quercetin reduced the melt viscosity but had a limited effect on electrical conductivity compared to alizarin, and the finest fibers containing this additive (achieved by adding 2% (*w*/*w*) liquid quercetin) were 36.72 µm in diameter. The analysis of fibers produced by melt spinning, melt spinning with post-drawing, and melt electrospinning revealed that the melt-electrospun fibers had a similar degree of crystallinity to PFs and are not comparable to drawn filaments.

## Figures and Tables

**Table 1:** Chemical structures and melting points of the dyes alizarin, hematoxylin and quercetin.

Dye	Chemical Structure	Melting Point [°C]
Alizarin	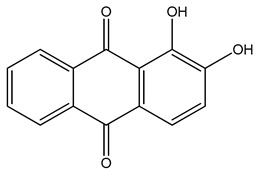	279–283
Hematoxylin	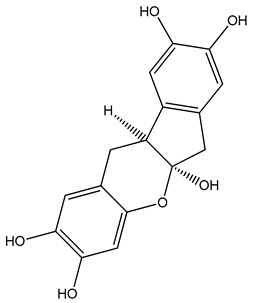	200
Quercetin	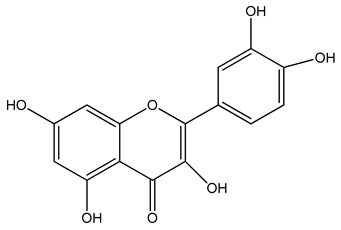	320

**Table 2:** Compound abbreviations according to the dye and weight percent.

Compound Abbreviation	Dye	% (*w*/*w*) of Dye	% (*w*/*w*) of Plasticizer
A1	Alizarin	1	0
A2	Alizarin	2	0
LA1	Liquid Alizarin	0.25	0.75
LA2	Liquid Alizarin	0.5	1.5
H1	Hematoxylin	1	0
H2	Hematoxylin	2	0
LH1	Liquid Hematoxylin	0.25	0.75
LH2	Liquid Hematoxylin	0.5	1.5
Q1	Quercetin	1	0
Q2	Quercetin	2	0
LQ1	Liquid Quercetin	0.25	0.75
LQ2	Liquid Quercetin	0.5	1.5

**Table 3:** Properties of PLA fibers, comparing partially orientated and drawn filaments with melt-electrospun fibers.

Material	T_g_ [°C]	T_cc_ [°C]	T_m_ [°C]	X_c_ [%]
Partially oriented PLA filament	62.90	95.70	174.10	25.26
Drawn PLA filament	–	–	175.70	58.83
Melt-electrospun PLA fiber	62.10	108.90	174.30	8.87

## Abbreviations

A	compounds containing alizarin
DF	drawn filament
DSC	differential scanning calorimetry
GPC	gel permeation chromatography
H	compounds containing hematoxylin
HFIP	hexafluor-2-isopropanol
LA	compounds containing alizarin and liquid plasticizer
LH	compounds containing hematoxylin and liquid plasticizer
LQ	compounds containing quercetin and liquid plasticizer
M_n_	number average molar mass
M_w_	molecular weight
PDI	polydispersity index
PF	partially oriented filament
PLA	polylactic acid
POM	polarized optical microscopy
Q	compounds containing quercetin
T_cc_	cold crystallization temperature
T_g_	glass transition temperature
TGA	thermogravimetric analysis
T_m_	melting point
X_c_	percentage crystallinity
